# Immunity Depletion, Telomere Imbalance, and Cancer-Associated Metabolism Pathway Aberrations in Intestinal Mucosa upon Short-Term Caloric Restriction

**DOI:** 10.3390/cancers13133180

**Published:** 2021-06-25

**Authors:** Evan Maestri, Kalina Duszka, Vladimir A. Kuznetsov

**Affiliations:** 1 Department of Biochemistry and Urology, SUNY Upstate Medical University, Syracuse, NY 13210, USA; evanmaes@buffalo.edu; 2 Department of Biology, SUNY University at Buffalo, Buffalo, NY 14260, USA; 3 Department of Nutritional Sciences, University of Vienna, Althanstrasse 14, 1090 Vienna, Austria; kalina.duszka@univie.ac.at; 4 Bioinformatics Institute, Biomedical Sciences Institutes A*STAR, Singapore 13867, Singapore

**Keywords:** duodenum mucosa, caloric restriction, gene expression, oncogenic pathways, immune response, telomere maintenance, metabolic reprogramming, cancer

## Abstract

**Simple Summary:**

Dietary restriction regimens, such as caloric restriction (CR), in the initiation and development of cancers has been studied using biological models and traditionally considers CR as anti-cancerogenic. However, the experimental, clinical facts and conclusions are controversial. CR-induced molecular and cellular mechanisms and pro-oncogenic pathways have not been systematically studied, leaving therapeutic benefits unclear. Here, using systems biology and deep data analysis approach, we study the CR-induced molecular pathway switches and cell-type context-specific responses known to underly early pre-malignant states in mouse and human mucosa. We identify the genes and energy-restricted networks associated with pre-malignant state metabolic reprogramming in normal stem cells and epithelial cell cycle activation, leading to telomere ends misbalance and immune response depletion. We define the changes in tumor suppressor and oncogenic pathways which may precede intestinal mucosa lesion development. This work will aid in the near future to define critical biomarkers for earlier detection and risk of adenomas and colorectal cancer.

**Abstract:**

Systems cancer biology analysis of calorie restriction (CR) mechanisms and pathways has not been carried out, leaving therapeutic benefits unclear. Using metadata analysis, we studied gene expression changes in normal mouse duodenum mucosa (DM) response to short-term (2-weeks) 25% CR as a biological model. Our results indicate cancer-associated genes consist of 26% of 467 CR responding differential expressed genes (DEGs). The DEGs were enriched with over-expressed cell cycle, oncogenes, and metabolic reprogramming pathways that determine tissue-specific tumorigenesis, cancer, and stem cell activation; tumor suppressors and apoptosis genes were under-expressed. DEG enrichments suggest telomeric maintenance misbalance and metabolic pathway activation playing dual (anti-cancer and pro-oncogenic) roles. The aberrant DEG profile of DM epithelial cells is found within CR-induced overexpression of Paneth cells and is coordinated significantly across GI tract tissues mucosa. Immune system genes (ISGs) consist of 37% of the total DEGs; the majority of ISGs are suppressed, including cell-autonomous immunity and tumor-immune surveillance. CR induces metabolic reprogramming, suppressing immune mechanics and activating oncogenic pathways. We introduce and argue for our network pro-oncogenic model of the mucosa multicellular tissue response to CR leading to aberrant transcription and pre-malignant states. These findings change the paradigm regarding CR’s anti-cancer role, initiating specific treatment target development. This will aid future work to define critical oncogenic pathways preceding intestinal lesion development and biomarkers for earlier adenoma and colorectal cancer detection.

## 1. Introduction

Calorie restriction (CR), where test animals receive a reduced energy diet, is one of the most broadly acting regimens for preventing or reversing weight gain and inhibiting cancer in experimental tumor models [1,2]. Protocols typically involve 10 to 40% reductions in total energy intake compared to ad libitum-fed controls, but with adequate nutrition and a controlled physical environment [2]. Chronic reduction of dietary energy intake without malnutrition decreases adiposity, inflammation, and improves metabolic profiles [3,4]. CR was shown to increase tumor latency and have protective effects in some experimental mammary carcinogenesis models [5,6]. Upon CR, metabolic alterations foster health-promoting characteristics including increased insulin sensitivity, decreased blood glucose and growth factors (IGF-1), and angiogenesis [4]. Reducing IGF-1 and glucose levels may decrease tumor progression [2,3,4,7]. Large bodies of experimental preclinical models support CR as anti-tumorigenic [2,3,4,7]. Yet, data have also demonstrated neutral and negative effects [8,9,10]. For instance, mice small intestinal response had a highly-dispersal trend decreasing large polyps (>2 mm) but increasing small polyp (≤2 mm) numbers [8]. CR started early in life reduced incidence/delayed progression in most rodent tumors, however, CR started in middle-aged mice had higher lifetime incidences of lymphatic neoplasms [10]. Whether CR results in protective or deleterious effects on cancer risk and outcome depends on the length and restriction severity [6,7]. A dominating anti-cancer CR effect decreases tumor rate growth via inhibition of circulating systemic factors (e.g., hormones/growth factors) which stimulate cancer cell proliferation [5,11]. However, many cancer subtypes lack hormone/growth factor sensitive cells (e.g., high aggressive basal-like cancers). Sensitive tumor clone(s) may be targets of pro-oncogenic metabolic reprogramming and/or replaced with more aggressive CR-resistant ones.

Recent randomized clinical trials have been explored for potential CR anti-cancer properties [12,13,14]. These trials showed mixture effects of CR directly on tumor tissue growth, host immune cells response, and other tissue responses (e.g., adipocytes). However, these studies do not mechanistically support the anti-cancer role of CR on neoplastic processes as indicated by large bodies of empirical experimental model data. CR-mediated reduction in cancer cell proliferation is central to anti-cancer animal model studies. The clinical trial studies showed no negative energy/calorie restriction effects on cell proliferation marker Ki-67 in Barrett’s esophagus [13] and breast cancers [14]. In men with prostate cancer, presurgical weight loss showed CR-mediated Ki-67 upregulation [12]. Additionally, high overexpression of several periodic cell cycle genes, cancer-associated genes, and oncogenes have been found [12,14].

The duodenum mucosa (DM) is a useful biological model for the study of CR small intestine response [15]. After epithelial cells, the most numerous cells in the lamina propria are immune cells, mainly duodenal intraepithelial lymphocytes (IEL) [16,17], present at 9–50 IELs per 100 epithelial cells that vary in different medical and bacterial conditions [17,18]. The DM transcriptome model showed dichotomized differentially expressed genes (DEGs) response to CR, with metabolic genes upregulated and immune genes downregulated [15]. However, DM tissue-specific transcriptional network profiles in CR-mediated cancer biology mechanics were not studied. The intestinal mucosa epithelium is the most highly proliferative mammalian tissue and CR further enhances epithelial regeneration [19,20]. Precise balances control the quiescent (G0-phase) and active intestinal stem cells, progenitors, and stroma cells. In healthy mice, short-term CR reduced cellular mass resulting in 15% shorter villi and reduced numbers of more differentiated epithelial progenitor cells. The proliferative rate and Lgr5 (high)/Olfm4+ active intestinal stem cell and Paneth cell numbers were modestly increased upon CR [21]. CR did not impact enterocyte apoptosis. Isolated crypts from CR mice in vitro can form primary and secondary organoid bodies with increased proliferation rate and intestinal stem cells and Paneth cells growth per crypt [21]. Cycling intestinal stem cells exhibit high Wnt activity, with CR sensitizing them to DNA damage [21,22]. This suggests CR initiates microenvironmental/stroma-mediated control loss, activates independent proliferation, and stimulates stem cell organoid body formation progression: cancer hallmark factors.

The direct CR anti-cancer and pro-cancer mechanics in normally proliferated intestinal mucosa are poorly understood. Systematic analysis of CR mechanisms in cancer biology has not been carried out, making therapeutic benefits unclear [12,23,24]. The contradictive tumor biology CR data and recent failed clinical trials [12,14] motivated us to use hypothesis-testing and data-driven system biology analysis of gene expression profiles changes in small intestine mucosa tissue upon short-term CR. We hypothesize that the CR response of the cell types in mucosa tissue may be the subject of cancer metabolic reprogramming mechanisms affecting oncogenes, cell cycle, immune response, chromosome maintaining genes, and pathways in cells responding to nutrient stress [25,26,27,28,29].

Our major objective is to identify cancer driver genes and oncogenic pathways induced by CR-related perturbation of cellular homeostasis affecting normal proliferation of epithelium mucosa via metabolic reprogramming and depletion of immune system control. Our central hypothesis is that CR activates metabolic reprogramming pathways in DM defined by cancer hallmarks (e.g., uncontrolled cell proliferation, tumor suppressors loss, etc.), while suppressing immune system surveillance; this may initiate occurrence or preferential competition of abnormally proliferating cells leading to pre-cancer and cancer risks. To test this hypothesis, we developed unbiased and comprehensive data analysis approaches. We use the mouse mucosa response to the 2-week 25% CR model in DM. Our results demonstrated CR induces drastic gene expression and pathway suppression of intracellular immunity and immune responses of T-, B-, NK- cells, macrophages, and their precursors. The family of immunity-related GTPases, including homologs of human Crohn’s disease susceptibility gene *IRGM*, were downregulated. We identify and characterize CR DEGs specifying regulatory networks modulating telomere stability and tumor suppressors, activating proliferative tissue-specific oncogenes, and chemical carcinogenesis. New CR response genes, prospective treatment onco-targets, and cancer risk factors are discussed. Our CR-induced metabolic reprogramming and multi-cellular competition models suggest plausible dysregulation of genes, networks, and pathways of pre-malignant and malignant states.

## 2. Materials and Methods

In this study, we carried out data analysis of differentially expressed genes (DEGs) of 26,966 signal values of probe sets (p.s.) from Affymetrix MoGene 1.0 ST microarray data (Affymetrix, Santa Clara, CA, USA) from mouse mucosa scrapings produced in our previous study [15]. The significant DEGs subsets in mouse DM samples of CR vs. ad libitum (AL) control mice was defined by expression microarray DEGs selected in common cases by the adjusted *p*-value <0.05 at |FC| > 1.5 and for telomeric DEG subset at |FC| > 1.2. Notations: x: microarray hybridization signal value in CR data, y: microarray hybridization signal value in AL data, Fold change/ratio score: if x > y then FC = x/y, if y > x then FC = −y/x, adj. *p*-value: adjusted *p*-value. Using these statistical criteria, 521 p.s. were selected. 16 p.s. without gene annotations were excluded. We determined 505 p.s. representing CR mice DEGs, with 467 unique annotated gene symbols (Data S1A,B). The gene annotation available for mouse MGI DB (mm9/GRm38 assembly, http://www.informatics.jax.org/marker/, accessed date: 1 May 2019) and microarray datasets were mapped using BLASTN and BLASTP to the mouse genome and transcriptome respectively, manually curated, re-annotated, and in some cases annotated de novo. We analyzed gene sequences and annotated gene symbols associated with multiple p.s. We analyzed normal human duodenum gene expression profiles [30] using statistical methods (Suppl. Methods Section S5) and studied gene expression correlations of mouse and human DM. We integrated other datasets using bioinformatics recourses (Suppl. Methods).

### 2.1. Computational Resources

Gene enrichment subset analysis, networks, pathways, collections of DBs, list publications used during manual curation of genes, and our selection criteria and statistical methods are described in Suppl. Methods. In this study, we systematically used Ingenuity Pathway Analysis (IPA) tools [31]. For the annotation, GO terms, and network connectivity enrichment analysis we also used: DAVID Bioinformatics 6.7 (https://david-d.ncifcrf.gov/), STRING v11 (https://string-db.org), Enrichr DB (https://amp.pharm.mssm.edu/Enrichr/). These tools were accessed starting 1 May 2019. A generalized systems biology protocol and workflow for bioinformatics, network analysis, databases, and tools utilized in this paper is available at dx.doi.org/10.17504/protocols.io.btqfnmtn accessed date: 1 May 2019.

### 2.2. Immune System and Epithelial Related Gene Curation

Initially the CR DEGs were separated using DAVID Bioinformatics Functional Annotation Tool selecting for enriched tissues jejunal and colic lymph nodes, spleen, activated spleen, and thymus. This produced a list of immune system-enriched genes which we further characterized using IPA resources, providing essential immune annotations. To extend our curation and mining, classification of the CR DEGs was completed using ImmPort gene lists available at https://www.immport.org/shared/genelists (accessed: 1 June 2021), including chemokines, TNF family members, interleukin receptors, and NK cell markers. We profiled the CR-responded immune DEGs using the Single Cell Portal (https://singlecell.broadinstitute.org/, accessed: 1 June 2021) from the Broad Institute. We visualized single-cell RNA-seq data of the murine small intestine epithelium (SCP241, GSE106510). This strengthened our classification system of the immune system genes by the cell-types: macrophage, CD8, DC, epithelial, plasma cell, inflammatory monocyte, neutrophil, CD4, NK, B cell, and pDC.

Epithelial-enriched genes were identified in the CR DEGs using DAVID Bioinformatics Functional Annotation Tool selecting for annotations colon, liver, kidney, and SI. Expression data from Paneth cells isolated from mice intestinal stem cell crypts on CR diet (60% of calories of ad libitum) diets for 4–7 weeks were downloaded from GSE37209 [21]. The adj. *p*-value cut-off for the Paneth gene list was 0.05. Differential expression data were obtained by *t*-test, as implemented by limma, and corrected for false discovery rate. Log2-transformed microarray signal intensity values were analyzed. GO analysis of the CR duodenum DEGs also commonly regulated in CR isolated Paneth cells was conducted. Single-cell RNA-seq data (SCP44, GSE92332) of the murine small intestinal epithelium were used to enhance the cell-type specificity to epithelial-annotated genes. A total of 50 highly expressed CR DEGs with epithelial annotations were profiled across the cell-types (including mature and progenitor): enterocyte, tuft, goblet, enteroendocrine, stem, Paneth.

### 2.3. Cancer Related Gene Curation

We curated genes lists from colorectal adenoma and duodenal adenoma/adenocarcinoma datasets, the Cancer Cell Metabolism Gene Database (https://bioinfo.uth.edu/ccmGDB/), Cancer Predisposition Gene (CPG) Database, Tumor Suppressor Gene (TSG) Database (https://bioinfo.uth.edu/TSGene1.0/), Cancer Stem Cell Database (CSCdb), OncoMX (http://oncomx.org/), dbEMT (http://dbemt.bioinfo-minzhao.org/) Epithelial-Mesenchymal Transition Gene Database, Apc knockout and APCMin/+ mouse model of colorectal cancer studies, and literature searches. These tools were accessed starting 1 May 2019. See Suppl. Methods Section 11 for details on full curation steps.

### 2.4. qPCR Data Analysis across Gastrointestinal (GI) Tract Tissues

We used qPCR data analysis for identification and validation of the CR-induced response of the DM, jejunum, stomach, ileum and proximal and distal colon. To characterize the response of DM and the entire GI tract to CR, we carried out qPCR analysis of 18 DEGs annotated in the DM microarray data. The genes represent epithelial mucosa cells metabolism, cancer-associated, and immune system genes including several hubs and essential cross-talk nodes linking CR functional sub-networks (see Results Section 3.12). Six parts of the GI tract tissues were analyzed, with 7–14 samples extracted from each tissue. Details about the RNA isolation from intestinal scrapings samples, genes, PCR primers, and quantitative PCR (qRT-PCR) reaction are presented in our publications [15,32] and Suppl. Methods Section 6.7. To carry out the univariate and multivariate analyses, we categorized qPCR gene expression values of each tissue into three categories: −1 if the CR induced significant down-reduction of a gene expression (decreases mean value), 0 if the gene expression variation is not significant, and +1 if the CR induced significant up-regulation of a gene expression (increases mean value). We also included the microarray-defined DEGs represented by the fold enrichment of the 18 genes.

## 3. Results

### 3.1. Analysis of Differentially Expressed Genes in Mouse Duodenum Mucosa

In our previous study, we carried out gene microarray expression profiling and its experimental validation for CR-responded DEGs in mouse DM [15]. In this work, we reanalyzed the microarray dataset using advanced bioinformatics and systems biology approaches to test our central hypothesis. In CR mice compared to ad libitum mice a total of 240 p.s. were significantly upregulated and 265 p.s. were downregulated; there are 222 upregulated unique gene symbols and 246 downregulated unique symbols. Data S1 indicates CR DEG categories.

Enrichment analysis of tissue-associated proteins was completed using the Uni-Prot datasets (Up_Tissue) annotation database version Sept 2009 (https://www.uniprot.org/, accessed: 1 June 2018). Through the selection of tissues with Benjamini <0.05, the Enriched Immune System Gene (ISG) subset was represented by 171 genes (132 downregulated, 39 upregulated) that referred to the jejunal and colic lymph nodes (at 87.14-fold enrichment), spleen, activated spleen, and thymus. Epithelial Cell-Enriched Genes (ECG) were represented by 152 genes (52 downregulated, 100 upregulated) that referred to the colon, liver, kidney, and SI. Additional immune resources [33] and IPA’s Disease and Biological Function annotations strengthened the classification system. The DEGs not included in the previous categories were called “Other Genes”. GO Analysis via DAVID Bioinformatics of the CR DEGs (Data S2) revealed top biological processes and pathways including immune response (*p* = 5.45 × 10^−7^) and glutathione transferase activity (*p* = 2.93 × 10^−9^).

### 3.2. DM Tissue Expressed Genes Are Translatable between Mouse and Human

**Figure 1:**
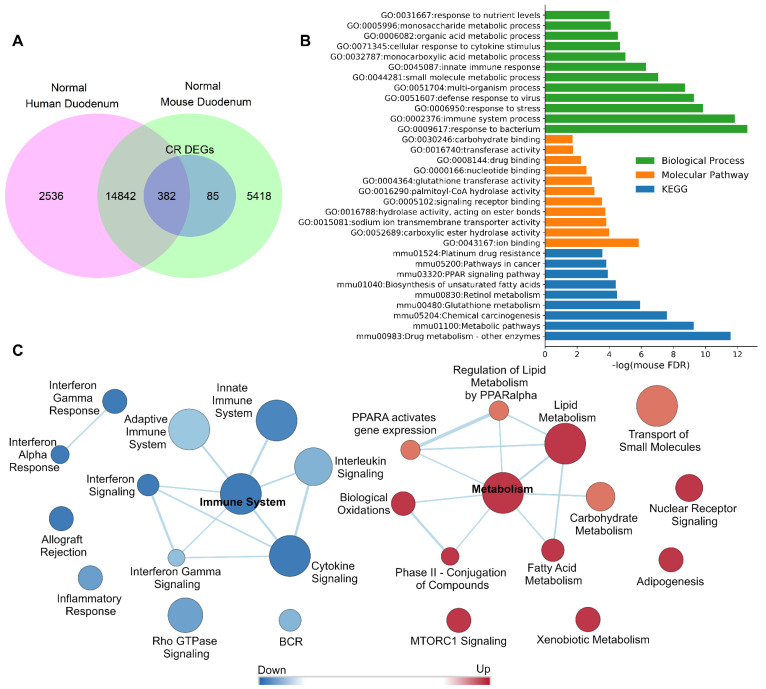
Association between mouse and human ortholog gene sets significantly expressed in normal mouse and normal human DM tissues and GO functional enrichment analysis of the mouse gene subset with human orthologs. (**A**) Venn diagram of mouse and human orthologous gene sets significantly expressed in normal mouse and normal human DM tissues. Normalized log10 transform microarray expression data for mouse and human DM tissues were analyzed (Suppl. Methods). A gene of a human or mouse dataset was considered expressed with a mean log signal intensity value larger than the cut-off value of three. (**B**) GO functional enrichment analysis of mouse DM DEGs with human orthologs responding to CR (genes selected from mouse significantly expressed gene subset of Panel A) were analyzed using the STRING v11 tools at enrichment FDR < 0.05. CR induced DEGs at adj. *p*-value < 0.05 and |FC| > 1.5. GO analysis of the 382 annotated CR induced mouse DEGs with human orthologs yielded 255 significant Biological Process (BP) terms. Top terms included the response to bacterium (GO:0009617, 44 genes), immune system process (GO:0002376, 78 genes), response to stress (GO:0006950, 103 genes), and defense response to virus (GO:0051607, 21 genes). The mouse CR DEGs were enriched in 67 Molecular Function (MF) terms and 34 KEGG Pathways including drug metabolism—other enzymes (mmu00983, 19 genes), metabolic pathways (mmu01100, 60 genes), chemical carcinogenesis (mmu05204, 15 genes), glutathione metabolism (mmu00480, 11 genes), and PPAR signaling pathway (mmu03320 10 genes). The same results with high confidence GO categories were found for the human orthologous genes in the common subset of Venn diagram Panel A. (**C**) Results from GSEA pre-ranked analysis (Suppl. Methods) of mouse CR DEGs (adjusted *p*-value < 0.05 at |FC| > 1.2). Gene sets (*p* < 0.05, FDR < 0.25) were visualized by Cytoscape Enrichment Map.

The translatability between experimental models was analyzed by comparison of microarrays data from the CR mouse duodenum to normal human duodenum expression (Figure 1A) [30]. We found 17,760 expressed genes in the normal human duodenum and 20,727 in the normal mouse duodenum (Suppl. Methods Section 5). The number of shared orthologous expressed genes between normal human and mouse duodenum was 15,224; this represents 85.72% (15,224/17,760) of the entire human DM expressed genes and 73.45% (15,224/20,727) of the entire mouse DM expressed genes. Additionally, 382 of the 467 mouse CR DEGs had human orthologs. High levels of similarity exist between the duodenum gene lists for mice and humans. GO and pathway analysis via STRING v11 determined the functional associations between the human and mouse orthologous genes. Figure 1B and Table S1 present the top enriched GO terms and pathways for the 382 CR DEGs with human orthologs. Gene set enrichment analysis (GSEA) indicated a dichotomization response in mouse CR microarray gene expression (metabolic and inflammatory) which we previously confirmed by qPCR [15] (Figure 1C).

### 3.3. 26% of CR Response Genes Are Involved in Mucosa Normal-Adenoma-Carcinoma Differential Gene Expression Patterns

We carried out a detailed meta-analysis of several datasets including human duodenal adenoma/adenocarcinoma, colorectal adenoma, duodenal cancer (Familial Adenomatous Polyposis cases) transcriptome data, and cancer cell metabolism genes. Our analysis also included APC knockout data, Ingenuity Pathway Analysis (IPA) functional annotations, PubMed and Google literature search, and manual literature curation.

**Figure 2:**
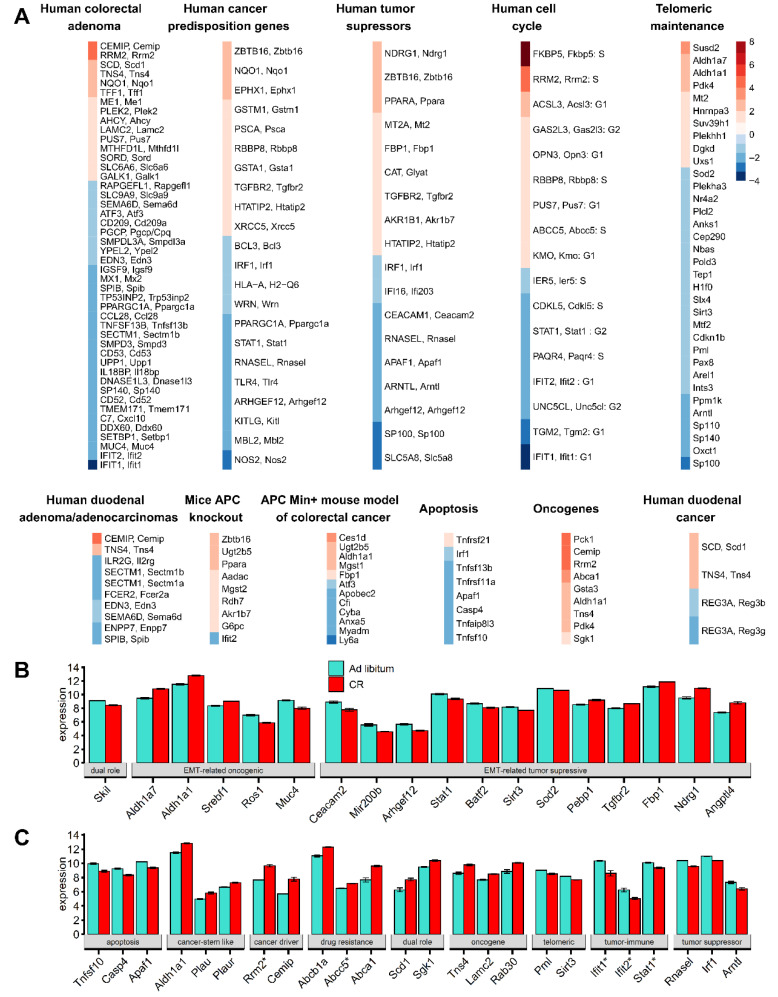
Categorization of CR-induced DEG subsets and their heatmaps. (**A**) When human/mouse gene orthologs are both present as labels, this indicates the original meta dataset used was human. The heatmap is colored by the gene expression fold change (FC) in CR DM mice (orange up, blue downregulated gene). Only matching directionality gene expression changes were included when comparing to the 505 CR mice DEGs (upregulated in both mice CR model and human colorectal adenoma, duodenal cancer, and duodenal adenoma/adenocarcinoma or both models downregulated). Data S3 lists the exact FC in previous models. Similarly, matched directionality gene expression changes for mice Apc knockout/APC^Min/+^ upregulated and CR mice downregulated or Apc knockout/APC^Min/+^ downregulated and CR upregulated was included. Human tumor suppressors were all suppressed in colon adenocarcinoma. Human cancer predisposition genes, telomeric maintenance, human cell cycle, apoptosis, and oncogene directionality in cancer models is not indicated. (**B**) Data are presented as the mean ± SEM (adjusted *p*-value < 0.05) for EMT-related CR DEGs with oncogenic, tumor-suppressive, or dual roles. (**C**) Selected CR DEGs presented as the mean ± SEM (adj. *p*-value < 0.05) representing key proliferative, oncogene, tumor suppressor, apoptotic, stem-like epithelial, tumor-immune surveillance, and telomeric genes which may play negative roles in the mucosa. *Sgk1* and *Scd1* (CR-upregulated) play dual roles in cancer progression, however, their upregulation promotes tumor growth and migration for colorectal carcinoma [43] and gastric cancer [44]. Genes with asterisks indicate cell cycle classification.

Data S3 and Suppl. Methods describe the full curation processes. Figure 2A shows the metadata analysis of duodenal pre-cancerous adenoma/adenocarcinoma patterns as regulated in CR mice. We determined a collection of 121 oncogenes, tumor-associated and cancer-related genes (Data S1R) which comprise 26% of our 467 mouse CR DEGs (adj. *p*-value < 0.05 at |FC| > 1.5), with 63 upregulated genes and 58 downregulated (Data S1S,T).

For instance, Data S3I–L lists the 20 DEGs genes of CR DM that have been observed in the APC^Min/+^ mouse model of colorectal cancer [34,35]. Of these 20 genes, 12 were upregulated (*Mgst2*, *Aadac*, *Ppara*, *Zbtb16*, *Rdh7*, *Ugt2b5*, *Akr1b7*, *G6pc*, *Aldh1a1*, *Mgst1*, *Ces1d*, and *Fbp1)* and eight were downregulated (*Ifit2*, *Ly6a*, *Myadm*, *Cyba*, *Apobec2*, *Atf3*, *Anxa5*, and *Cfi*) in CR mice. These findings suggest a co-regulation pattern of CR DEGs and Apc knockout genes/the APC^Min/+^ mouse model of colorectal cancer [34,35]. They are relevant to our hypothesized mucosal response to CR via aberrant tumorigenic regulatory processes.

Next, we compared CR response genes found in mice DM with the DEGs of normal-adenoma-carcinoma patterns [30,36,37,38]. Human duodenal adenoma/adenocarcinoma and colorectal adenoma transcription expression data (orthologs with corresponding mouse CR DEGs matched regulation directionality changes) indicated 53 significant cancer-associated genes of the 467 CR DEGs: 15 upregulated genes, 38 suppressed (Data S3A–F).

Using the Cancer Predisposition Gene Database [39], 24 human orthologues with corresponding mouse CR DEGs were identified as cancer predisposition genes (CPGs), genes in which inherited mutations in confer increased cancer risk: colon cancer CPGs (*GSTM1*, *MBL2*, *RBBP8)* and gastric cancer CPGs (*NOS2*, *PSCA*, *TLR4*).

The Tumor Suppressor Gene Database (https://bioinfo.uth.edu/TSGene/) includes 535 human tumor suppressor genes (TSGs) with lower expression in colon adenocarcinoma samples compared to normal tissue samples [40]. Eight TSGs were downregulated upon CR (*Rnasel*, *Irf1*, *Ifi203*, *Sp100*, *Slc5a8*, *Apaf1*, *Arntl*, *Arhgef12*) and nine TSGs were upregulated upon CR (*Ppara*, *Tgfbr2*, *Ndrg1*, *Mt2*, *Htatip2*, *Zbtb16*, *Glyat*, *Akr1b7*, *Fbp1*). Using the CheEA3 transcription factor (TF) enrichment analysis tool, we observed that transcription of seven (*Ppara*, *Tgfbr2*, *Ndrg1*, *Mt2*, *Htatip2*, *Akr1b7*, *Fbp1*) of the nine up-regulated DEGs genes (called group 1 TSGs) could result in TF binding sites (BS) for liver X receptors (LXRs) (GEO-GSE35262, ChIP-Seq). In contrast, transcription of six (*Rnasel*, *Irf1*, *Ifi203*, *Sp100*, *Slc5a8*, *Apaf1*) of the eight upregulated DEGs (called group 2 TSGs) could result from TF-BS CDS2 interactions binding to promoters of mouse intestinal villus (GEO-GSE34566, ChIP-Seq). Upregulation of group 1 TSGs have alternative functions referring to activation of lipid metabolisms, while inhibition of group 2 TSGs is directly involved in villus morphology, cell organization, and developmental functions. Our DM villus model suggests CR-induced TSG function reduction/loss.

We also asked if there is a risk of highly aggressive and drug-resistant tumors associated with CR-responded DEGs in DM epithelial cells. Figure 2B shows that using the dbEMT, an epithelial-mesenchymal transition (EMT) associated gene resource [41], CR DEGs were classified as: oncogenic EMT-related genes (CR upregulated: *Aldh1a1*, *Aldh1a7*, *Srebf1*; CR downregulated: *Ros1*, *Muc4*) and tumor-suppressive EMT-related genes (CR downregulated: *Ceacam2*, *Mir200b*, *Arhgef12*, *Stat1*, *Baft2*, *Sirt3*, *Sod2*; CR upregulated: *Angptl4*, *Ndrg1*, *Fbp1*, *Tgfbr2*, *Pebp1*). *Skil* (suppressed upon CR), was classified as an EMT-related gene with dual roles (oncogenic and tumor-suppressive function). These findings suggest that CR may induce a risk of aberrant EMT to stemness, aggressiveness, and cancer state [42]. Outcomes of such balance depend on chromosome instability, including telomeric and cell cycle pathways.

### 3.4. Telomeric Maintenance Pathways and Cell Cycle Gene Responses in CR Mice

A total of 34 telomeric CR mice DEGs (adj. *p*-value < 0.05 at |FC| > 1.2) were collected from the databases TelNet and IPA, literature searches, and manual curation of TERC, TERRA network, and DNA damage/repair gene sets (Data S1Q and Data S4, Suppl. Methods Section 11). Overall, 70.6% (24/34) telomeric DEGs were suppressed in CR mice, indicating telomeric maintenance dysregulation and chromosomal instability.

To identify CR DEGs directly involved in the cell cycle, we searched for mouse orthologues in CycleBase comprehensive human cell cycle periodic genes lists (Table S2). Due to CR in mice, nine DEGs were upregulated (*Fkbp5*, *Rrm2*, *Acsl3*, *Gas2l3*, *Opn3*, *Rbbp8*, *Pus7*, *Abcc5*, *Kmo*). Enrichment analysis (STRING v11) showed that Rrm2 and Rbb8 form a functional network with Brca1 and Atm suggesting their involvement in homologous recombination. Additional associations: Rbb8 (G2/M DNA damage checkpoint) and Rrm2 (P53 signalling, glutathione pathways). Eight downregulated cell cycle CR mice DEGs (*Ifit1*, *Ifit2*, *Stat1*, *Tgm2*, *Unc5cl*, *Pagr4*, *Cdkl5*, *Ier5*) refer to immune system suppression, inflammatory, and immune cells development. Gene associations included positive cellular response to innate immune response (interferon-alpha, interferon beta) (*Ifit1*, *Ifit2*, *Stat1*; FDR < 0.025) and I-kappa-B kinase/NF-kappa B signalling regulation (*Stat1*, *Unc5cl*, *Tgm2*; FDR < 0.005), which dysregulation of occurs in chronic inflammatory diseases and certain cancers [45].

Figure 2C indicates genes with potential for substantial negative roles in CR DM mucosa.

### 3.5. Mice Mucosa Cellular Immune System Compartments Are Deeply Suppressed by CR and Epithelial Network Interactions Are Activated

**Figure 3:**
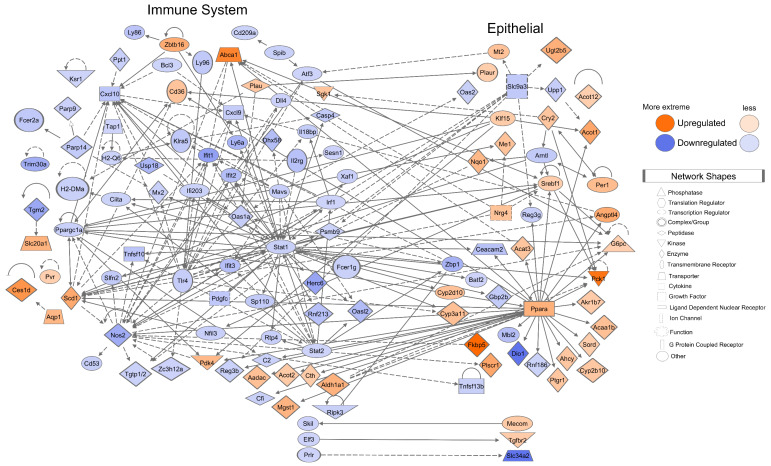
CR suppresses the immune system and preferentially activates epithelial cell DEG networks and discriminates the networks interconnection. IPA DB and network graphical tools were used to construct the immune system and epithelial cell-specific subsets DEGs and their encoded protein-protein interactions. 174 edges (protein interactions) were found among high significantly connected immune network interactions with several super hubs (e.g., Stat1 with 36 edges and Tlr4 with 19 edges). CR reduces the expression of a vast majority of the immune genes. CR modulates the expression of epithelium cells, activating metabolic genes, which had 58 edges within epithelial-specific proteins (e.g., Ppara pathway directly connected with 26 edges). A total of 34 edges were from immune to epithelium interactions (with a hub of Stat1, 7 edges), and 45 edges were from epithelium to immune interactions (with hub Ppara, 16 edges, mostly linked with the upregulated immune system proteins). The blue color node indicates downregulated proteins and the orange color node indicates upregulated proteins. Color intensity is based on the gene expression fold changes. Bold lines indicate a direct relationship. Dotted lines indicate an indirect relationship.

Upon CR, strong/global immune system suppression and epithelial activation occurred (Figure 3). The 171 ISGs consist of 37% (171/467) of CR-responding DEGs. *Stat1* (immune) and *Ppara* (metabolic) drivers induce de novo abnormal regulation of DM pathways. The immune and epithelial network contained 118 proteins encoded by CR DEGs, 311 edges (protein interactions), average number of neighbors 3.64, clustering coefficient 0.14, network density 0.02, and PPI network enrichment *p* = 1.00 × 10^−16^. Subcellular ISG and ECG localization networks are in Figure S1. Single-cell profiling and annotations of the immune and epithelial DEGs are in Figure S2. 

**Figure 4:**
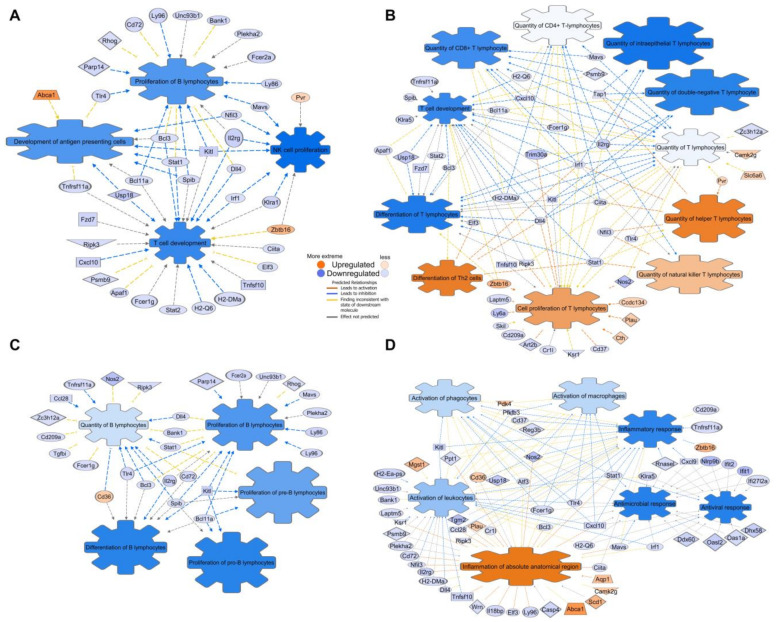
CR reduces T-, B-, NK- and antigen-presenting cells expression, their functional activity, and suppresses interconnections between major immune cell type populations. (**A**) IPA network analysis reveals global suppression of the major immune cell types. (**B**) T and NK lymphocytes network. (**C**) B lymphocyte network. (**D**) Inflammatory cells network. The Path Designer in IPA colors the disease/functional annotations and edges based on their current predicted effects; orange is predicted activation, blue is predicted inhibition, yellow is findings inconsistent with the state of the downstream molecule, and grey color is effect not predicted.

The suppressed ISGs were characterized using the Disease and Biological Function annotation tool from IPA (Table S3). The Lymphoid Tissue Structure and Development categorization (*p*-value dynamical range: 4.07 × 10^−19^ – 2.94 × 10^−3^) included 66 DEGs within its categories. Figure 4A shows the functional annotations proliferation of B lymphocytes (*p* = 9.52 × 10^−10^, *n* = 18), T cell development (*p* = 5.12 × 10^−9^, *n* = 24), development of antigen presenting cells (*p* = 7.18 × 10^−7^, *n* = 10), and NK cell proliferation (*p* = 7.31 × 10^−6^, *n* = 8) displayed as a network with 31 proteins encoded by CR DEGs. 

A total of 45 T cell-associated functional annotations (Figure 4B) and 27 B cell-associated functional annotations were displayed (Figure 4C). Additionally, 60 Inflammatory Response DEGs (*p*-value dynamical range: 2.47 × 10^−16^ – 2.94 × 10^−3^, *n* = 77) were selected (Figure 4D). An additional suppressed ISG functional annotation network is provided in Figure S3. Our analysis determined a deep suppression of cellular immune system genes belonging to all tissue-associated lymphocyte populations and immune system regulatory cells, suggesting systemic immune-cell-specific quantity reduction in the mucosa.

### 3.6. CR Induces Downregulation of Embryonal/Haematopoiesis/Immune CSC Genes, However Upregulation of Epithelial Cell CSC Genes

Cancer stem cells (CSCs) are a subpopulation of cancer cells possessing characteristics associated with normal stem cells, specifically self-renewal and differentiation giving rise to the cell types found in a particular cancer sample [46]. CSCs are tumorigenic (tumor-forming), perhaps in contrast to other non-tumorigenic cancer cells. The CSCdb (http://bioinformatics.ustc.edu.cn/cscdb) provides the annotations of 74 marker genes of 40 different CSC lines and 1769 CSC-related genes [46]. Table S4 indicates 17 CSC-related genes and 1 marker CSC differentially expressed upon CR, with *Aldh1a1* identified as both. Overall, eight CSC DEGs were upregulated including epithelial cell-type associated (*Plau*, *Plaur*, *Aldh1a1*, *Bnip3*, *Mecom*, *Mgst1*, *Abcb1*, *Ndrg1*), and 10 were downregulated, mostly genes related to embryonic, haematopoiesis, and immune cells (*Ly6a*, *Kitlg*, *Il2rg*, *Dll4*, *Mecom*, *C2*).

### 3.7. Functional Characterization of the Common Subset of DM and Paneth Cells DEGs

To specify the epithelial cells associated with CR-induced DEGs, we carried out the comparison of the common DM (Data S1A) and Paneth cell DEGs that responded to CR in mouse mucosa (Data S1V) [21]. We downloaded and analyzed 1656 Paneth cell DEGs (*p* < 0.05). We identified 90 common DEGs.

High similarity of the gene profiles common between Paneth cell DEGs and DM DEGs was found (*r* = 0.426, *p* < 0.001, Spearman). By Wilcoxon Signed Rank Test, the null hypothesis is highly accepted (*p* = 0.68; two-sided). Friedman’s ANOVA test provides a similar result (*p* = 0.91; two-sided). Most of the common 90 genes were up-regulated in both datasets due to CR. Only eight genes (8.9%) were down-regulated in Paneth cell DEGs and 19 (21.1%) genes were down-regulated in DM DEGs. The frequency difference was significant (*p* = 0.022, by the test of Ratio of Two Binomial Proportions).

GO enrichment analysis showed the common DEGs belong to STRING protein-protein local networks of metabolic pathways (*p* = 1.6 × 10^−6^; mmu01100, KEGG; p = 1.8 × 10^−14^, MMU-1430728; Reactome Pathways), retinol metabolism (mmu00830), fatty acid, monocarboxylic, sodium ion, and oxidation-reduction process. We also identified high enrichment of DEGs involved in chemical carcinogenesis (mmu0504; KEGG Pathways; *p* = 6.2 × 10^−7^) which are linked to oxidation-reduction sub-networks via Ppara. Additional functional analysis categorization included abnormal xenobiotic pharmacokinetics (Slc47a1, Gstm1, Abcb1a), abnormal basal metabolism (Per1, Cd36), increased circulating ketone body level (Pdk4, Cd36, Scd1), decreased glycogen level (Pdk4, Cd36, Ppara), and abnormal intestinal mucosa morphology (Angptl4, Abcb1a, Enpp7).

Of the 34 common DM and Paneth DEGs included in our annotation of cancer-associated genes, the vast majority (28 of 34, 82.4%) were upregulated. Tumorigenesis risk is associated with upregulated periodic cancer cell cycle genes (*Fkbp5*, *Acsl3*) and enzymatic mutagenesis genes (*Cy3a44*). Only upregulated DEGs associated with telomeric maintenance (*Susd2*, *Pdk4*, *Aldh1a1*, *Mt2*, *Aldh1a7*) were observed in the common Paneth and DM DEG list. Most of the immune system genes in the common gene subset (17 of 23; 74%) were upregulated. The list of 90 common genes did not include tumor suppressors and apoptosis genes; however, we identified six strong up-regulated pro-oncogenes/oncogenes (*Aldh1a1*, *Sgk1*, *Rab30*, *Abca1*, *Tns4*, *Cemip*).

### 3.8. Gene Ontology and Pathway Analysis Revealed That 26% of CR Responded DEGs Are Involved in Malignancy and Cancer–Associated Networks

**Figure 5:**
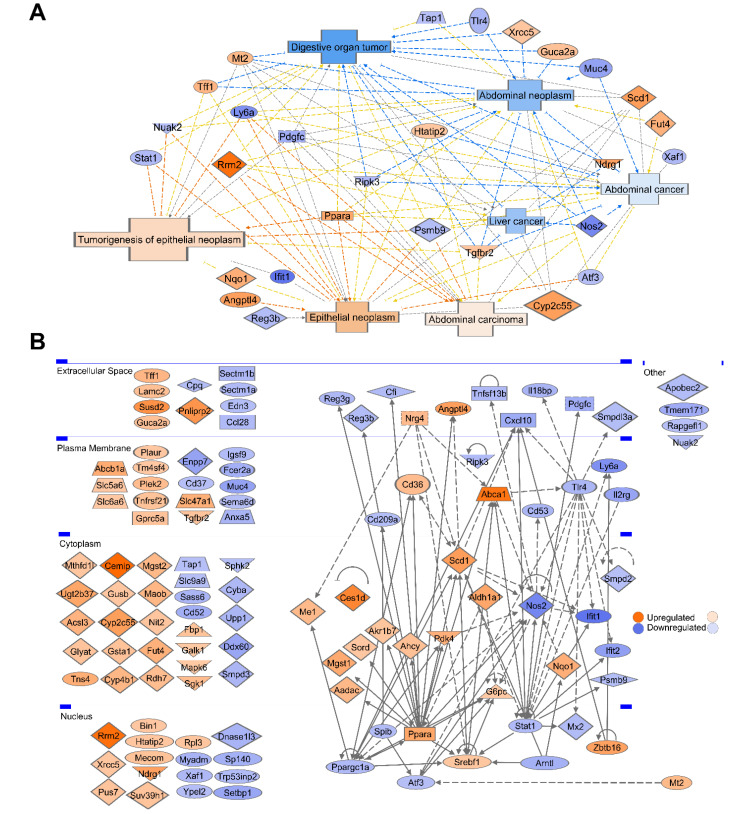
Cancer subcellular network and disease functional annotations. (**A**) A network of 28 proteins based on the functional annotations of the cancer-associated DEGs. (**B**) The network includes 43 connected and 78 non-connected cancer-associated proteins encoded by DM genes distributed across four cellular compartments: extracellular space, plasma membrane, cytoplasm, nucleus. Top hubs include Ppara (25 downstream targets, 6 upstream targets), Stat1 (17 downstream, 12 upstream), Ppargc1a (12 downstream, 8 upstream), Tlr4 (12 downstream, 1 upstream), Scd1 (10 downstream, 7 upstream), and Nos2 (8 downstream, 13 upstream). These hubs account for 54.1% (131/242) of the edge connectivity.

The 26% (121/467) of DEGs were characterized in IPA (Table S5). Within the Cancer category (*p*-value dynamical range: 7.03 × 10^−12^ – 2.64 × 10^−3^, *n* = 48) disease annotations with predicted activation included abdominal carcinoma (*p* = 7.96 × 10^−8^, *n* = 12), epithelial neoplasm (*p* = 2.40 × 10^−7^, *n* = 20), and tumorigenesis of epithelial neoplasm (*p* = 1.08 × 10^−4^, *n* = 13) (Figure 5A). Annotations with predicted inhibition included abdominal neoplasm (*p* = 7.03 × 10^−12^, *n* = 23), digestive organ tumor (*p* = 1.63 × 10^−10^, *n* = 21), abdominal cancer (*p* = 3.00 × 10^−10^, *n* = 17), and liver cancer (*p* = 7.57 × 10^−8^, *n* = 11). This indicates CR has downstream potential for both pro- and anti-cancerogenic effects. Upregulated pro-oncogenic genes associated with malignant condition and down regulated tumor suppressor genes are listed in Table S6. We highlight future qPCR validation targets of CR in cancer-initiation mechanism contexts (Table S7). For example, upregulation of CEMIP, RRM2, LAMC2, SCD, TNS4, and PLAU in humans is prognostically unfavorable for various cancers [47]; these genes have CR-upregulated mouse orthologs.

Among the 121 DEGs, 43 proteins had subcellular location forming a cancer-associated network (Figure 5B) containing 121 edges, average number of neighbors 3.67, clustering coefficient 0.15, network density 0.05, and PPI network enrichment *p* < 1.00 × 10^−16^.

### 3.9. Network of the Cancer-Associated, Immune System, Epithelial, and Telomere Gene Subsets

Enriched canonical pathways were generated through IPA for each gene subset (Table S8). The Sirtuin Signaling Pathway was enriched in: 467 unique DEGs (*p* = 1.38 × 10^−3^, *n* = 15), cancer-associated (*p* = 1.32 × 10^−6^, *n* = 11), telomeres (*p* = 9.77 × 10^−5^, *n* = 5), ECGs (*p* = 8.51 × 10^−4^, *n* = 8). We highlight the Sirtuin Signaling Pathway because it had second highest significance for the cancer-associated network canonical pathways (Table S9). Transcription of only one of the seven sirtuins, Sirtuin 3, was significantly reduced.

**Figure 6:**
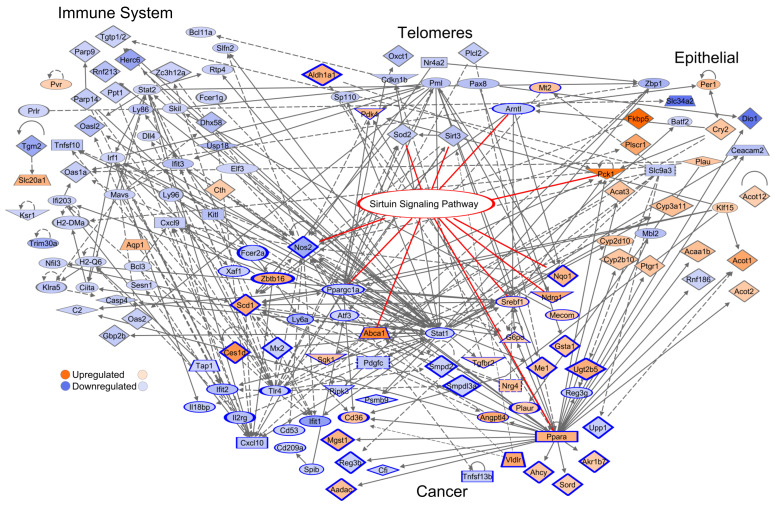
Cross-talk networks with Sirtuin Signaling Pathway. Cancer-related proteins have a dark blue shape outline. Sirtuin Signaling Pathway proteins have red edges. All DEGs encoding these proteins have adjusted *p*-value < 0.05 at |FC| > 1.5, except the telomere subset with |FC| > 1.2. Top hubs include Stat1 (44 downstream targets, 25 upstream targets), Ppara (42 downstream, 7 upstream), Tlr4 (25 downstream, 4 upstream), Ppargc1a (19 downstream, 11 upstream), Nos2 (14 downstream, 20 upstream), Pml (10 downstream, 4 upstream), and Cxcl10 (1 downstream, 17 upstream). These hubs account for 34.7% (243/700) of the edge connectivity.

The immune system, cancer-associated, epithelial, and telomere subsets were displayed as a network with the Sirtuin Signaling Pathway overlaid (Figure 6). The network had 132 connected components, 227 non-connected components, 350 edges, average number of neighbors 3.74, clustering coefficient 0.14, network density 0.02, and PPI network enrichment *p* < 1.00 × 10^−16^. Figure S4 shows the network interactions of the tumor-immune and tumor-epithelial microenvironments identifying possible mechanisms of CR-induced malignancy risks or anti-cancerogenic effects.

### 3.10. CR Induces a Network between DEGs of Glutathione, Chemical Carcinogenesis, and Sirtuin Signaling Pathways

**Figure 7:**
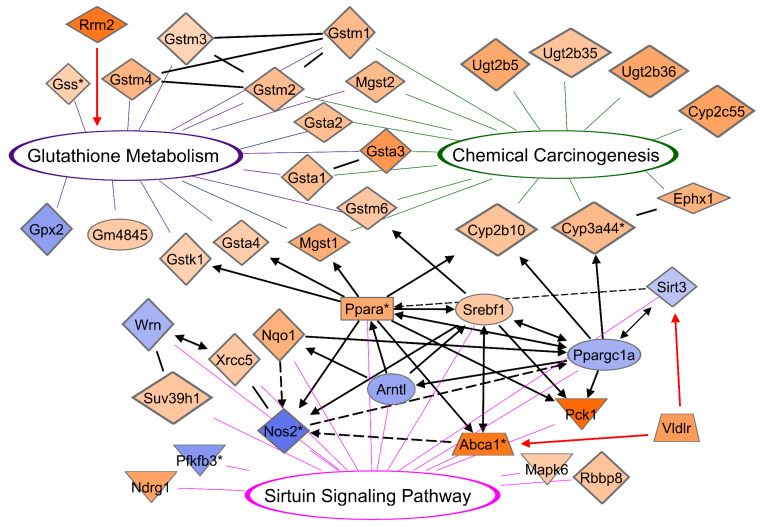
CR induces a network between proteins encoded by DEGs of glutathione, chemical carcinogenesis, and sirtuin signaling pathways. DEGs of glutathione metabolism (purple; mmu00480), chemical carcinogenesis (green; mmu05204), and sirtuin signaling pathways (magenta). Sirtuin signaling regulation has downstream targets in glutathione metabolism (Gstk1, Gsta4, Mgst1, Gstm6) and chemical carcinogenesis (Cyp2b10, Cyp3a44). Black lines indicate molecule relationships. Self-loops were removed for easier visualization of network interactions between molecules. Asterisks on molecules indicates druggable targets: Ppara (aleglitazar, atorvastatin/choline fenofibrate, bezafibrate, clofibrate, docosahexaenoic acid, ezetimibe/fenofibrate/simvastatin, gemfibrozil, NS-220, pemafibrate, TPST-1120, tesaglitazar), Nos2 (GW 273629, N(G)-monomethyl-d-arginine, pimagedine, triflusal), Cyp3a44 (atazanavir/cobicistat/darunavir), Abca1 (probucol), Gss (*N*-acetyl-l-cysteine), Pfkfb3 (PFK-158). All DEGs encoding these proteins are adjusted *p*-value < 0.05 at |FC| > 1.5, except Sirt3 with FC-1.38.

Glutathione metabolism (FDR = 1.20 × 10^−6^, *n* = 11), chemical carcinogenesis (FDR = 2.53 × 10^−8^, *n* = 15), and sirtuin signaling (*p* = 1.38 × 10^−3^, *n* = 15) gene subsets are highly enriched in our DEG dataset [48,49]. Consistent with these findings, IPA enrichment analysis revealed significant functional interconnections between proteins encoded by DEGs of glutathione, chemical carcinogenesis, and sirtuin signaling pathways (Figure 7). With one down-regulated exception (Gpx2, blue), the interconnected DEGs are upregulated (orange) in glutathione/chemical carcinogenesis pathways. This network model supports SIRT3 as a central coordinator of CR-mediated metabolic reprogramming of DM cellular composition. We included RRM2 in our model, a cell cycle protein (CycleBase-3) involved in the glutathione metabolism pathway (KEGG). VLDLR was also included in our model as a protein encoded by an upregulated metabolism gene *Vldlr* upon CR with dual functions in lipid metabolism and as a pro-oncogene in gastric cancer, breast cancer, liver adenocarcinoma, and other cancers [50,51].

### 3.11. CR Profiles Common between Duodenum and Liver

We analyzed the short-term 3-week CR liver response at 75% of the diet as ad libitum feeding (GSE51885) [52]. This revealed 55 genes commonly regulated in duodenum and liver upon short-term CR with 49/55 genes (89.1%) regulated with matched directionality fold changes in both tissues (Figure S5). Of these genes, several of our hypothesized duodenal key cancer-risk or immune modulators were also regulated in the liver: suppressed immune and inflammatory response (*Stat1*, *Ifit1*, *Cxcl10*), activated metabolism including glutathione (*Ppara*, *Gsta2*), tumor suppressor loss (*Arntl*), and activated oncogenes (*Fkbp5*, *Rab30*, *Mt2*). Careful attention to tissue-specific oncogenic changes and risks for metabolic reprogramming should occur in future CR analysis of other tissues.

### 3.12. DM Microarray-Defined DEG and GI Tract Tissue Samples qPCR Analysis Suggests Correlated Positional Expression Patterns of CR-Induced Response along the GI Tract

To compare CR-induced DEG patterns in mucosa of the different GI tract tissues, we selected 18 DM DEGs which respond to CR representing epithelial cell metabolism, immune system cells and pro-oncogenic/cancer-associated expression genes. Table S10A shows the list of the DEGs for immune system (*Stat1*, *Tlr3*, *Irf1*, *Reg3b*, *Reg3g*, *Oas1a*) and metabolism (*Acot4*, *Acox2*, *Scd1*, *Cd36*, *Pparα*, *Vldlr*, *Gsta3*, *Gsta4*, *Mgst1*, *Mgst2*) defined by qPCR in stomach, duodenum, jejunum, ileum, proximal and distal colon [15,32]. Six parts of the GI tract tissues were analyzed, with 7–14 samples extracted from each tissue. The genes include several hubs and essential cross-talk nodes linking CR functional sub-networks (e.g., *Pparα*, *Stat1*, *Scd1*). We also included in the list our qPCR expression data for *Mt2* and *Myd88* (Suppl. Methods). In total, the 18 gene expression profiles for the six GI tissue types were compared with the DEG expression patterns from microarray DM data.

Kendall’s tau paired correlation coefficients (Statistica, version 13.5) between qPCR expression data for stomach, jejunum, ileum, proximal and distal colon, and microarray DM fold change data showed that 15 of the 16 correlation coefficients were positive at *p* < 0.05 and one correlation coefficient (microarray DM FC vs stomach qPCR) is positive, but it is borderline significant (Table S10B). The strongest correlation pairs are D-J, J-I, D-I, PC-DC, J-PC, and J-DC which form a compact cluster. Microarray DM and qPCR DM are also highly correlated, suggesting that the duodenum, jejunum, ileum, proximal and distal colon DEGs form co-regulated network expression and pathways common for CR-response across the GI tract compartments of the mice mucosa tissue. Qualitatively similar results were found after calculation of Pearson’s correlation coefficients.

**Figure 8:**
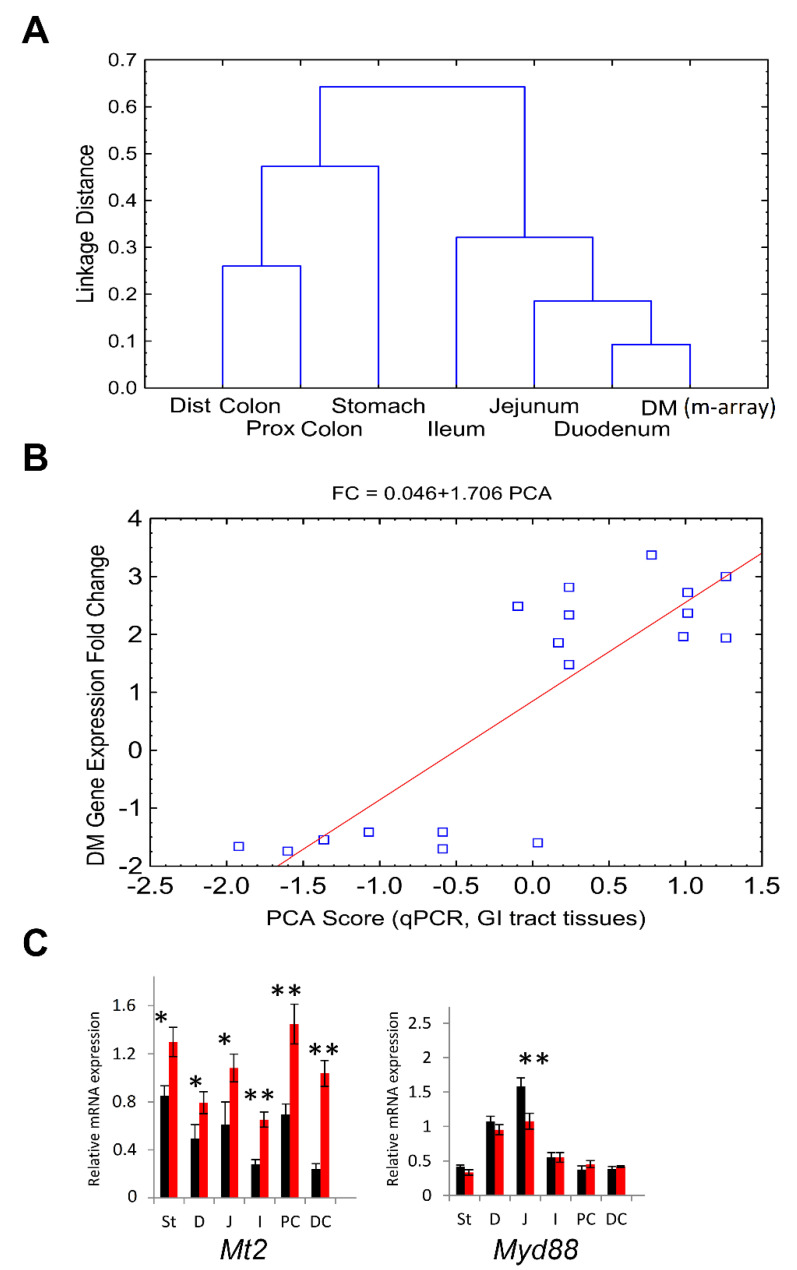
Comparing GI tract tissues responses to CR defined by qRT-PCR. (**A**) Hierarchical cluster analysis, showing two distinct GI tract clusters after calculating the distance between tissues in the CR qPCR response of 18 genes. (**B**) Principal component analysis (PCA) of the six gastrointestinal (GI) tract tissues. (**C**) Relative mRNA expression of *Mt2* and *Myd88* measured in six tissues along the GI tract. Two-tailed Student’s *t*-tests were used to assess statistical significance; *n* = 7–14, * *p* < 0.05, ** *p* < 0.01. St-stomach, D-duodenum, J-jejunum, I-ileum, PC proximal colon, DC distal colon. CR induces significant over-expression of *Mt2* across all studied tissues. *Mt2* is one of a few immune system genes upregulated upon CR in DM. The qPCR-defined expression pattern of *Myd88* shows it is likely not sensitive to CR among five of six GI tract compartments.

Hierarchical cluster analysis (Figure 8A) shows that duodenum (qPCR, microarray), jejunum and ileum form one group, but distal and proximal colons and stomach form another group.

Principal component analysis (PCA) of all qPCR detected GI tracts samples (Figure 8B) showed that the 1st principal component explained 72% of tissue sample variability (*p* = 3.7 × 10^−9^). Spearman correlation between the microarray DM DEG fold change (FC) and qPCR 1st principal component score values of the 18 genes was high (*r* = 0.77, *p* < 0.001).

Figure 8C shows representative examples (*Mt2*, *Myd88*) of the robust qPCR-defined expression patterns across the GI tissues that were differentially expressed in our microarray data. Tumorigenic and immune modulation functions of these genes were considered in the Suppl. Methods Section 14.

Thus, our results showed the consistency of CR-induced gene expression patterns defined in both DM and qPCR in different parts of the GI tract mucosa. The microarray DM was a representative tissue for our study of CR phenomena. Additionally, two distinct DEG patterns (duodenum, jejunum, ileum) and (stomach, proximal and distal colon) could be considered.

### 3.13. Interferon-Inducible DNA-Binding Gene Family Members on Chr 11 Are CR-Suppressed

**Figure 9:**
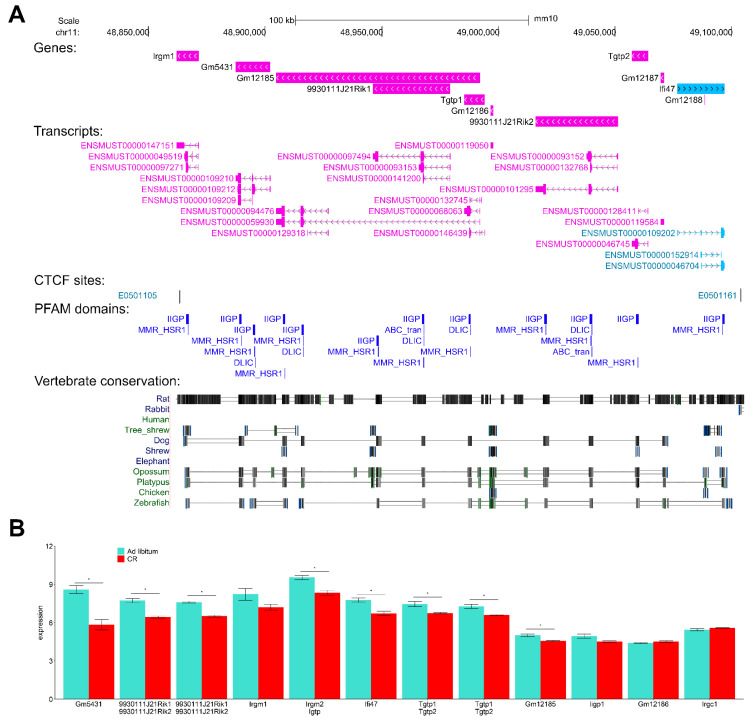
Chr11qB(1.2) locus comprising 10 genes belonging to the CR IFN-induced family. (**A**) Genes and their transcribed isoforms are annotated by the latest Ensembl (Build 75) currently supported by the UCSC genome browser (Build 100 annotations in Data S5). All PFAM annotated proteins have one or more conserved functional domains. Pfam-A annotates transcript regions within the peptide recognizable as Pfam protein domains in characterized and non-characterized protein-coding genes for mouse assembly mm10 track of UCSC by the software HMMER3: IIGP (Interferon-inducible GTPase; PF05049), MMR_HSR1 (50S ribosome-binding GTPase; PF01926), DLIC (Dynein light intermediate chain; PF05783), ABC_tran (ABC transporter; PF00005). CTCF sites are displayed by the ENCODE Candidate Cis-Regulatory Elements (cCREs) track combined from all cell types. We used the UCSC “Multiz alignments” track, mouse assembly mm10, with multiple alignment data combining PhyloP and PhastCons methods. It shows evolutionary conservation from the PHAST package for 60 vertebrates and three subsets (Glires, Euarchontoglires, placental mammal). (**B**) Data are presented as the mean ± SEM. Asterisks indicate adj. *p*-values < 0.05 for statistical significance.

One of the top CR DEGs, predicted/uncharacterized protein-coding gene 5431 (*Gm5431*) localized in Chr 11qB(1.2), was strongly suppressed (FC = -6.76; *p* = 2.70 × 10^−17^). We observed that the gene belonged to Chr11qB(1.2) locus containing a family of paralog genes that encode interferon (IFN)-inducible GTPase proteins, also called immunity-related p47 GTPases (INF-I GTPases). Interferons induced intra-cellular programs functional in innate and adaptive immunity against infectious pathogens. Chr 11qB(1.2) locus comprises *Gm5431* with genes *Irgm1*, *Tgtp1*, *Tgtp2*, *Ifi47*, and uncharacterized genes *9930111J21Rik1*, *9930111J21Rik2*, *Gm12185*, Gm12186, and Gm12187 (and their transcribed isoforms) (Figure 9A and Data S5). In total, 9 of the 10 genes were localized on the chromosome negative strand. Genes in this locus had high evolutionarily conserved sequences and paralogous in several other loci (for instances, Chr 11qB(1.3) (*Irgm2*, *Igtp*), Chr18 (*Iigp1*, *F830016B08Rik*)), in other chromosomes and orthologous in genomes of many species (Figure 9A and Figure S6). IRGM in humans is an ortholog to this family, with links to Crohn’s disease, inflammatory disorders, and non-alcoholic fatty liver disease [53]. *Irgc1* (with RefSeq transcript NM_199013) located on Chr 7 is also an ortholog of the gene family to the human genome (IRGC) but its transcription was not significantly regulated by CR (Figure S6).

Using MAFFT-DASH-multiple alignment software integrated with structural search [54], we found high confidence similar sequences and domains between IIGP1 and all our proteins (Data S5). Longer proteins encoded by *Gm5431*, *9930111J21Rik1*, and *9930111J21Rik2* showed the highest sequence similarity with IIGP1/1TQ4 and included two non-overlapping IIGP1 sequence homologous. IIGP1, the member of INF-I GTPases encoded by gene *Iigp1* localized on Chr18, has known crystal structure and atom resolution 3D model 1TQ4. IIGP1/1TQ4 is built of two domains, the G domain and a helical domain including five DNA-binding motifs [55].

Due to high sequence similarity and the presence of evolutionarily conserved sequence domains (Figure 9A), we suggest structural and functional similarity of IIGP1 and the members of the INF-I GTPase family analyzed in this section.

The transcription levels of the IFN-I-GTPases were commonly suppressed due to CR response (Figure 9B). Additionally, the Chr. 11qB(1.2) loci is flanked by CTCF binding sites. Analysis of the IFN-inducible gene family members suggests that CR in DM induces global suppression of interferon-induced cellular functions in innate and adaptive immunity.

### 3.14. Drug Targets in Energy-Responsive Metabolic Pathways

A total of 36 drug targets were associated with the 467 mouse duodenum CR DEGs as identified by IPA (Data S6). PPARα, NOS2, TLR4, and CXCL10 are the most relevant strongest hubs with the highest connectivity determined by network analysis. TLR4 is targetable by eritoran, GSK1795091, OM 174 lipid, and resatorvid. The activity of CXCL10 is modulated by MDX-1100. Anti-CXCL10 monoclonal antibodies have implications in infectious disease, chronic inflammatory, and autoimmune disease therapeutics and attenuate murine inflammatory bowel disease and murine AIDS colitis [56]. Agents that deplete cellular glutathione metabolism in combination with arsenic trioxide are studied for the treatment of non-acute promyelocytic leukemia [57]. Attempts to increase the efficacy of cancer treatments via lifestyle or diet changes would benefit from further examination of these top targets in nutrient-responsive pathways.

## 4. Discussion

Because pre-malignant and cancer cells must reprogram their metabolic state in every step of progression to survive in nutrient-altered conditions, metabolic reprogramming is recognized as a cancer hallmark [58]. Defining how cancer cells rewire metabolism towards proliferation, which our understanding of has progressed rapidly, opens new therapeutic intervention strategies [25,26,27,28,29]. In our study, we for the first time identified cancer driver genes and genes playing key roles in oncogenic pathways induced by CR perturbation of cellular composition, tissue homeostasis, and metabolic reprogramming, leading to cell proliferation of epithelium mucosa and global depletion of immune system control.

We found that 26% of the CR responding differential expressed genes (DEGs) in mice DM consist of cancer-associated genes—most never studied in CR contexts. These responses may lead to perturbation of telomere maintaining mechanisms, and activation of the cell cycle, pro-oncogenic, and CSC pathways including EMT. CR-induces metabolic reprogramming, which affects the ISGs, consisting of 37% of the total DEGs; the majority of ISGs are suppressed, including cell-autonomous immunity and tumor immune evasion controls.

### Major Findings of This Study

1. CR induces tissue homeostasis dysregulation, shifting the transcriptome profile to pro-oncogenomic pathway patterns in DM associated with activation of metabolism and proliferative activity of epithelial cells including Paneth cells, but depletion of intracellular immunity and functions of all immune cell types;
2. CR induces transcription of cell cycle genes including a subset of key cancer-associated signaling genes directly involved in reprogramming pathways, premalignancy, malignancy states, and poor outcome in mice and humans;
3. In CR response, apoptotic gene expression is reduced or not significantly varied;
4. Tissue-specific proliferative epithelial stem DEGs are activated, however, immune-specific stem cell/progenitor genes are suppressed upon CR;
5. CR induces transcription activation of key tumor-susceptible and oncogenes gene sets and their networks with tissue-specific risk of carcinogenesis and down-regulates protective mechanisms mediated by key tumor suppressor genes;
6. CR induces multiple transcription suppression effects in autophagy, tumor-immune surveillance mechanics, and genes associated with induction and effector stages of NK, T-, B- cells and macrophages immune response;
7. Detoxifying exogenous chemicals enzymes and drug metabolism networks with glutathione pathways are activated upon CR but could be involved in anti-cancer and pro-cancer outcomes via mutagenesis and DNA damage/repair mechanisms.

**Figure 10:**
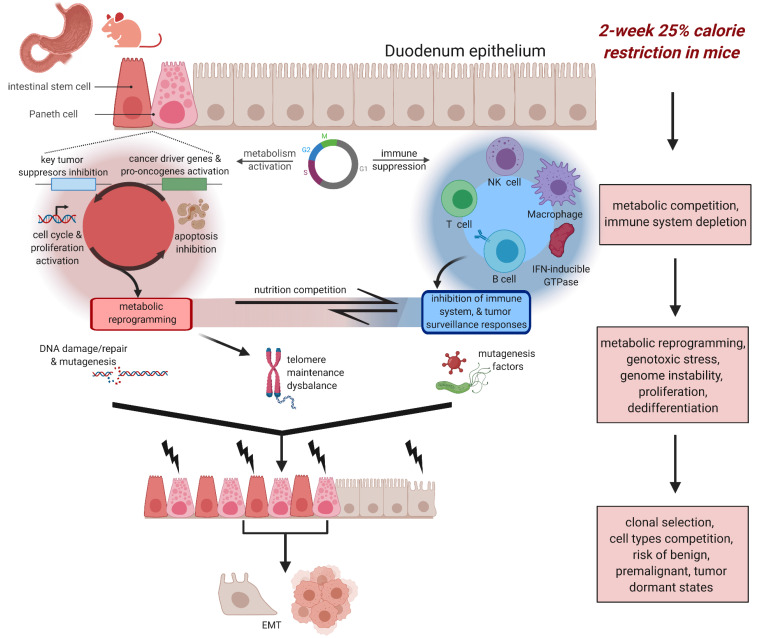
CR cellular disbalance models through immune and autophagy suppression. CR-induced metabolic reprogramming and immune suppression. DEGs and pathways analyses strongly support our hypothesis that CR dramatically changes the epithelial versus immune cells relationships. Functional interactions modulating the homeostasis cooperation between cell types may increase malignancy risk. Morphologically, mucosa enterocyte differentiation and their cell type density depend on the cell position along both the vertical axis (crypt-villus) and horizontal axis (proximal to distal) of the GI tract. At steady-state in normal conditions (homeostasis) of mucosa tissue, the cell types at differentiation states are position-dependent and relationships between cell types remain balanced. Our results suggest that CR induces a hypocellular and multiple cell-types disbalance by stimulation of cell proliferation and autonomation of this process. Our model suggests that CR response leads to metabolic activation, inducing cell cycle genes and proliferation of epithelial stem cells. CR reduces the total mass of epithelial cells (vertical axis) [21], autophagy, apoptosis mechanisms, and telomere stability. Immune cell populations including progenitors and effector cells are globally reduced, suggesting increased pathogen susceptibility. If the processes controlling DNA and RNA damage induced by chemical carcinogens become dysregulated, it increases the risk of transformation and uncontrolled proliferation of abnormal stem-like cells.

Our network and pathway analysis provide interconnections of CR DEGs with biological functions, molecular mechanisms, cell types, and cellular compartments. Figure 10 shows the two-compartmental response models which hypothesize possible dysregulation mechanics of tissue/cell homeostasis upon CR in intestinal mucosa tissue leading to pro-oncogene pathway activation and increased potential risk of premalignant and cancer states. Figure S7 shows key pathways and gene targets that may be involved in the multiple disbalances increasing the risk of metabolic reprogramming leading to malignant states. Table 1 shows the selected list of the CR-response genes that expression is strongly supported by literature data as key genes of oncogenesis, immune suppression, and metabolic reprogramming and could be considered and tested as CR-induced cancer risk markers.

CR induces the directed activation of metabolism (lipids, purine/pyrimidine, oxygen, glucose, etc.) in intestinal stem cells and Paneth cells, but generates resource deficiency and stress pathways in immune cells. CR is the stress factor in all immune system cells in DM that via metabolic re-direction/competition leads to immune system depletion. The induction of functional and metabolic activity of epithelial cells (e.g., Paneth cells) leads to proliferation and metabolic process of the stem cells. The genetic program and rate of the proliferative and dedifferentiation response provides a high preference for intestinal stem cells and Paneth cells in comparison to more mature epithelial cells and essentially suppresses immune cells and immunity mechanisms. By shifting the energetic demands towards metabolism, genes normally expressed in canonical metabolism pathways (e.g., *Fkbp5*, *Rrm2*, *Vldlr*, *Cemip*) upon CR become upregulated. For example, *Vldlr* is a pro-oncogene involved in both lipid metabolism, proliferation, and dedifferentiation [50,51]. Thus, there is a dual role of many of the metabolically-upregulated CR genes in cancer risk. This metabolic reprogramming may precede lesion development due to the modulations in pro-oncogenes, tumor suppressor genes, and EMT pathways in epithelial cells.

However, due to the large body of research supporting CR as anti-cancerogenic, we note that essential cancer-risk genes in our networks may require some additional stress signaling (via chemical or enzymatic directed mutagens, DNA damage, infection, telomere maintaining imbalance) to ever fully realize precancerous lesion development. Intestinal stem cells and Paneth cells may become highly sensitive to genotoxic stress, leading to telomere and other types of genome instability, the selection of abnormal and malignant clones with strong transcriptome disbalance, uncontrolled growth, loss of apoptosis mechanisms, and gain preference in the competition for metabolites.

Enhanced expression of very low density lipoprotein receptor (VLDLR) in the intestine has been associated with stimulated cell proliferation and cancer development [50,51], hence with an increased energy demand. CR DM shows strong upregulation of VLDLR which mediates epithelial lipid endocytosis [59]. This coincides with our results showing that the majority of CR-affected metabolic genes are connected with lipid metabolism. VLDLR is a representative example involved in the cross-talk pathways with its protein network partners linking telomeric maintenance/oxidative stress response (Sirt3), immune system response (Cd36), and Paneth cells (Abca1, druggable target). Under metabolic restriction, the intestine raises lipid metabolism-associated gene expression at the cost of an extinguished immune response. The switching on of metabolism-associated genes which may promote or precede cancer development and metabolic reprogramming is an inherent risk to CR.

**Table 1:** CR DEGs in tumor suppressive, oncogenic, immune, epithelial stem/progenitor, anti-cancer, and detoxifying pathways.

Gene Name	CR	FC	Annotation	References
*Fkbp5*	Up	8.54	most over-expressed CR-response gene, androgen-responsive gene with high expression in esophageal adenocarcinoma (EAC) tissues and this is associated with decreased patient survival, pro-oncogenic role in EAC	[60,61]
*Vldlr*	Up	2.81	pro-oncogene involved in both lipid metabolisms and proliferation, pathogenesis of gastric cancer, breast cancer, and involvement in cancer cell growth	[50,51,59,62]
*Tlr4*	Down	−1.77	innate immune system, pathogen recognition, therapeutic target, reduced expression associated with metastatic status of CRC	[63,64,65,66]
*Arntl*	Down	−1.87	tumor suppressor, circadian rhythms	[67,68,69,70]
*Plau*	Up	1.78	CSC, poor pancreatic ductal adenocarcinoma prognosis	[71]
*Ly6a, Ly6e, Ly6c1*	Down	−2.67, −2.18, −1.63	immune cell differentiation, cancer stem cell biology	[72,73]
*Sirt3*	Down	−1.38	oxidative stress, protection of DNA damage, chromosome maintenance	[48,49,74,75]
*Rnasel*	Down	−1.74	tumor suppressor, predisposition to prostate cancer, antiviral pathways	[76,77,78,79,80,81,82]
*Cxcl9, Cxcl10*	Down	−1.79, −2.03	proinflammatory chemokines, therapeutic target	[56,83]
*Ndrg1*	Up	2.65	tumor suppressor, EMT, therapeutic target	[84,85,86,87]
*Ugt2b5, Ugt2b35, Ugt2b36*	Up	2.5, 1.7, 2.56	detoxifying enzymes, reduce risk of carcinogenesis and toxicities by inactivating aromatic-like metabolites	[88]
*Cyp2c55 (and family)*	Up	2.70	metabolizing endogenous compounds, detoxifying exogenous chemicals, drug metabolism	[89]
*Lamc2*	Up	1.75	promotes proliferation, cell migration, and invasion in cancers including colorectal and malignant metastases	[12,47,90]
*Rrm2*	Up	4.02	cell cycle, therapeutic target, oncogene playing a key role in tumorigenesis and cancer progression, poor prognostic factor for colon, breast, and pancreatic cancers, cancer driver	[91,92,93,94,95]
*Aldh1a1*	Up	2.45	EMT-related oncogenic, CSC, upregulated in APC^Min/+^ mouse model of colorectal cancer	[96]
*Casp4*	Down	−1.87	apoptosis, CASP4-deficient mice exhibit a defect in autophagy	[97]
*Cemip*	Up	4.25	overexpression correlates with poorer colon cancer patient survival and facilitates colorectal and stomach tumor growth, cancer driver	[24,47]

Our findings indicate the multifunctional roles of Paneth cells involved in maintaining the balances between response to pathogens, CR and injury-inducible stem cell activity, carcinogenesis pathways, homeostasis, cell proliferation and differentiation [21,98,99,100,101]. Paneth cell specific genes observed in our CR DEGs list are associated with regulation of mTORC1 signals supporting stem cell proliferation and maintaining [21,101]. Thus, the metabolism activation at the level of the specific-cell subpopulation is partially explained by Paneth cells. Changes in the Paneth cell niche in the intestinal mucosa are heavily implicated in tumor formation [21,101,102,103]. The list of 90 common Paneth and DM CR DEGs genes includes upregulated *Pdk4* and *Cemip*. PDK4 over-expression is involved in cell transformation of colon epithelial cells and poor outcome of several cancers [104]. *Pdk4* is a target for *Ppara* and also considered as a target for cancer detection and therapeutic strategies [104]. Cell migration inducing hyaluronan binding protein (CEMIP, KIAAI199) contributes to cell migration, invasiveness, uncontrolled proliferation, dedifferentiation, EMT, dysregulation of cytokine pathways, and is associated with poor outcomes in CRC patients [24,105,106]. This gene plays a key role in the protection of the tumor from hypoxia and leads to enhanced ability of tumors by stimulation with Wnt and EGFR [106].

According to our model, long-term CR may confer increased tumorigenic risks if there are durable and sustained increases in stem mutations and abnormal proliferation, DNA damage, epigenetic modification, and cell numbers that can undergo mutagenesis. Activated stem cells of DM villi may exhibit reprogramming behavior if metabolic machinery is used at higher rates, in combination with aggressive chemical and pathogenic bacterial factors. While external to our analysis, bacteria (*H. Pylori*), parasites, and viruses may provide the mutagenesis factors initiating oncogenesis in mucosa epithelial cells. Pathogenic microbiota signals could also act on the intestinal stem cell niche and homeostasis upon CR [107].

The multiple gene dysregulation in mucosa upon CR response could be interpreted as an “early ischemia”-like syndrome observed in newborns, demonstrating marked reduction of villous height and increasing epithelium crypt-villus ratio (in the vertical axis) in combination with small numbers of immune cells in intestinal lymphoid structures along the lamina propria (horizontal axis) [98]. These changes may resemble those seen in autolysis. Reduction or no change in apoptosis gene expression in epithelial cells occurs and no acute inflammatory cells are activated [98]. This coincides with global INF-induced autophagy reduction in our study.

CR induces epithelial cell cycle proliferation and suppresses apoptosis, increasing cancer risk. We observed suppressed apoptotic genes upon CR. Fasting, short-term, and long-term dietary restriction almost uniformly reduces cellular proliferation (liver, bladder, skin, heart, colorectum) explaining anticarcinogenic effects of CR [5]. A potential pro-cancerogenic effect in fasted-refed animals resulted from proliferation increases with apoptosis decreases in response to refeeding [5]. CR in some experimental models enhances cell death rate, however, proliferation/apoptosis rates vary through the course of treatment [5,11,21]. CR enhances the proliferation of Lgr5+ intestinal stem cells, the cell-of-origin for intestinal precancerous adenomas, and leads to the first organoid and acceleration of the second organoid formations [11,21].

In normal physiological conditions, cell cycle and apoptosis gene activity in tissue-specific stem cells, progenitors, and differentiated epithelial cells maintain tissue homeostasis. However, short-term CR disturbs this balance and may switch on pro-oncogenic pathways. For instance, *Rrm2* is highly upregulated in DM CR response. RRM2 is an oncogene playing a key role in tumorigenesis and cancer progression, including colorectal and oesophageal cancers [91,92,93,94,95]. CR-activated cell cycle periodic genes in our study are directly or indirectly involved in tumorigenesis pathways, drive cancer cell proliferation, and aggressiveness (RRM2, ACSL3, RBBP8, KMO, FKBP5), drug resistance (ABCC5), and modulate chemosensitivity (FKBP5) (references in Table S2A). *Fkbp5* is the most over-expressed CR-response gene. This androgen-responsive gene has high expression in esophageal adenocarcinoma (EAC) tissues and is associated with decreased patient survival [61]. FK506-binding protein 5 (FKBP5) plays a pro-oncogenic role in EAC [60,61]. In tissue resection specimens from EAC patients, FKBP5 expression was positively associated with proliferation as measured by Ki-67 expression, which was also observed in the prostate cancer weight loss clinical trials [12,60]. FKBP5 is a cis-trans prolyl isomerase that binds to the immunosuppressants FK506 and rapamycin. The number of activated CR mice cell cycle DEGs and their expression level increments (fold change) were stronger in the upregulated genes vs down-regulated DEGs. Our model showed high upregulation of FKBP5 and RRM2 (FC = 8.5 and FC = 4.0 respectively) compared to downregulated TGM2 and PAQR4 (FC = 3.1 and FC = 1.76 respectively). *Tgm2* and *Paqr4* may provide suppressive effects in some aggressive cancer cells (Table S2B) [108,109]. TGM2-siRNA knockdown attenuated colorectal cancer cell growth through the wnt3a/β-catenin/cyclin D1 pathway [109].

The pioneering NCI-funded (R21 CA161263) randomized clinical trial of prostate cancer patients undergoing presurgical CR-mediated body mass intervention manifested significantly grated Ki67 proliferation rate results [12]. It determined activated DEGs related to tumor cell signaling associated with increased proliferation, transcription, oncogenesis, migration, and invasion: *CBLC*, *POLB*, *ATF1*, *TFEB*, *ACVR1B*, *LAMC2*, *GSK3B*, *PHF6*, *MAP3K8*, *ARIDIA.* Our study also demonstrated CR-induced *Lamc2* overexpression. LAMC2 promotes proliferation, cell migration, and invasion in cancers including colorectal and malignant metastases [12,90]. LAMC2 has been suggested as a therapeutic target because of its association with the Wnt/β-catenin signaling pathway and effects on PI3K and ACT [12,90].

Our model includes potential gene regulatory switches modulating anti-cancer to pro-oncogenic CR response. CR induces specific mechanisms involved in pathogenic or adaptative EMT-driven stem cell response and proliferation of mucosa epithelial cells. The EMT pathway gene expression alteration [42] may or may not indicate markers solely, but silencing/dormant states could be formed. Due to CR-induced immune system depletion and mutagenesis/genotoxic events (leading to occurrence and accumulation of a driver mutation and genome instability), the risks of oncogene activation and tumor suppressor depletion required for conversion to tumor-initiating cells over long periods could increase [110]. This suggests that tumor lesions may need to be studied in experimental systems over time with a lower threshold detection levels of small benign polyps and dormant malignant states [8]. Time course systems biology analyses of CR severity and length should be conducted on pre-existing tumor dormant states [5,11].

Oncogenes and pro-oncogenic genes are poorly studied in CR and carcinogenesis contexts. Searching in PubMed revealed only 32.2% (39/121) of our CR cancer-associated genes (Data S7) are associated with terms “caloric” or “calorie” or “caloric restriction” or “calorie restriction” or “dietary restriction”. Table S6 includes upregulated oncogenes upon CR: *Cemip*, *Tns4*, *Aldh1a1*, *Rab30*, *Rrm2*, and *Gsta3*. CEMIP mRNA overexpression correlates with poorer colon cancer patient survival and facilitates colorectal and stomach tumor growth (OncoMX) [24]. TNS4 expression is transcriptionally regulated by MAP kinase signaling pathway and plays a critical role in tumorigenesis in several tissues including the colon. TNS4 and ALDH3A1 expression levels were increased in HCT-8 colon cancer cells and influence cancer cell migration, invasion, and proliferation [111]. RAS oncogene family member 30 (RAB30) was upregulated in the microarray of epithelial colorectal adenocarcinoma and acute lymphoblastic leukemia-derived cell lines [112]. PPARα-sensitive genes during starvation include *Cxcl10* and *Rab30* [113]. *RRM2* plays oncogenic roles in tumorigenesis [91] and is a poor prognostic factor for colon, breast, and pancreatic cancers [92,93,94,95].

Glutathione (GSH) plays a dual role in cancer progression. The Glutathione S-transferases (GSTs), phase II detoxification enzymes, were activated in CR mice: membrane-bound microsomal (*Mgst1*, *Mgst2*) and cytosolic family members (*Gsta1*, *Gsta2*, *Gsta3*, *Gsta4, Gstm1*, *Gstm2*, *Gstm3*, *Gstm4*, *Gstm6*, *Gstk1*). In healthy cells, GSH is crucial for the removal and detoxification of carcinogens [99]. However, GSH metabolism is associated with colorectal cancer pathogenesis [114]. The GSH system regulates proliferation and survival by offering redox stability in a variety of cancers [115,116]. MGST1 is crucial for stem cell differentiation [117]. *MGST1* polymorphisms may contribute to CRC risk [118]. GST-overexpressing phenotypes are present in many drug-resistant tumors (including breast, colon, and lung cancers) [115]. We propose therapies targeting the GSH antioxidant system in tumors in combination with CR may sensitize cancer cells to treatment [99] and reduce the risk of cancerous effects in CR conditions. Mice treated with l-buthionine-sulfoximine depleted GSH levels in esophageal cancer and decreased tumor burden, inhibited cell proliferation, and activated cell apoptosis [119].

The Sirtuin Signaling Pathway mediates CR-responded cancer-associated networks. This pathway regulates cancer cell metabolic reprogramming in glucose-poor environments [74]. Pck1, a CR upregulated Sirtuin Signaling Pathway gene, is associated with cancer cell gluconeogenesis and increased PCK1 expression is crucial for cancer growth in the absence of glucose [120]. In our study, upon CR in mice DM, expression of *Sirt3* isoforms (probe set 10568997, NM_022433) was downregulated. Sirtuin 3 (SIRT3) is a major NAD(+)-dependent mitochondrial deacetylase and the key regulator of fundamental processes frequently dysregulated in aging and other diseases [48,49]. New experimental designs may be pivotal to validate SIRT3 as a cancer regulator in CR.

CR induces multiple transcription suppression effects in the genes of autophagy and tumor immune surveillance mechanics. In contrast to activation of the metabolic genes associated with epithelial cells, we found immune system transcription suppression in 37% of DEGs induced by CR. Restricted cell cycle genes in interferon-inducible pathways include antiviral and anti-cancer activity [121] (*Ifit1* and *Ifit2)* and pro-inflammatory reactions recruiting immune cells to target cells [122] (genes *Stat1* and *Unc5cl*) (Table S2B).

Negative role of CR in tumor-immune surveillance. The immune system classified CR-responding DEGs were majorly suppressed for T cells, B cells, and macrophages (Table S11). The systemic CR-induced suppression response pattern was also shown in CR-responded immune DEGs with human orthologs for TNF family members, chemokines, interleukin receptors, NK cells, and antigen presenting cells (Table S11H). An intact immune system is essential to prevent neoplastic cell development and progression [123,124]. Mice with a homozygous deletion of the Rag-2 alleles completely lack NK-like T, T and B cells and have increased incidence and growth of spontaneous tumors and chemically induced cancer lesions [125]. IFN-γ forms the basis of an extrinsic tumor-suppressor mechanism in immunocompetent hosts [123,124,126]. STAT1 can suppress tumor formation [123]. The elimination of interferon IFN-γ or STAT1 genes in mice resulted in increased incidence and growth of spontaneous and chemically induced tumors [123,125,126]. CR may impact IFN-mediated tumor elimination [127].

CR induces suppression of INF pathways in DM. Type II interferon signaling (IFN-γ) is suppressed by CR. IFN-inducible GTPases play central roles in defending the mammalian cell’s interior against a diverse group of invading pathogens. The IFN-inducible GTPases ability to control infection at the level of an individual cell—a process termed cell-autonomous immunity—is responsible for microbial killing [128]. We identified IFN-inducible GTPase genes on Chr11q B(1.2) which were downregulated upon CR. Irgm1-deficient mice have Paneth cell abnormalities with increased risk for acute intestinal inflammation [129]. *Ifit1*, *Ifit2*, *Ifit3*, *Ifi203*, *Ifi44*, *Ifi27l2a*, and *Irf1* (all CR-suppressed) function in antiviral and antimicrobial response. Ifit1 knockout promoted viral replication in murine norovirus infected cells [121]. Loss of interferon regulatory factor 1 (IRF1) function causes severe susceptibility to infections in mice and humans [130]. IRF1 suppresses tumor cell growth, stimulating immune responses against tumor cells [130,131,132,133]. Defects in *IRF1* are associated with gastric and lung cancer, and myelogenous leukemia [131]. The *Stat1/2* and *Irf1/9* CR-network interactions mediate immunity and gut inflammation [122].

Further discussion of key modulators of CR-response in metabolic reprogramming and immune suppression in Table 1 can be found in the Suppl. Discussion including tumor suppressor Rnasel (CR-downregulated), proinflammatory chemokines Cxcl9/10 (CR-downregulated), immune cell differentiation Ly6 gene family (CR-downregulated), predicted therapeutic target Ndrg1 (CR-upregulated), and detoxification enzymes (CR-upregulated).

In summary, our results suggest that CR-mediated metabolic reprogramming suppresses multiple host tumor surveillance prevention mechanics including cell-autonomous immunity and activates signaling pathways of pro-oncogenes, tissue-specific cycling, and silenced stem cells, and cancer predisposition genes driving pre-malignant and cancer states. This study’s limitations refer to meta-data sets available for analysis, data type (e.g., microarrays), DM expression profile composition (cellular mixture), CR experimental time design, and pre-clinical models of CR effects on tumor pathobiology. However, our systems biology, data-driven, and hypothesis-testing approach to metadata provides feasibility by identifying DM DEGs subsets according to cell-type specificity functions: IFN-inducible GTPases genes, CR-induced cancer- and immune system cross-talks. This provides multiple determinants of aberrant signalling, cell-cell interaction networks leading dynamically to cell transformation, clonal selection, immune response delay, immune surveillance suppression, and tumorigenesis [134].

These findings may change the paradigm regarding the anti-cancer role of CR and initiate new unbiased CR cancer biology studies. Clearly, no single cell type/subtype or unique pathway accounts for all anti-cancer or pro-oncogenic effects of CR in normal, but complex mucosa tissue. As with most chronic disease intervention strategies, combination approaches improving lifestyle (CR, diet, physical activity), prophylactic strategies, and reproducible pharmacological interventions that target specific cells and their pathways are needed to prevent pre-cancer and cancer states. Future (bioengineering) directions to implement our results is to identify novel drugs and CR mimetics, compounds that mimic the specific CR mechanism. This will allow protection against cancerous metabolic tissue reprogramming pathways, chromosome stability, provide normal stem cell differentiation and proliferation, ensure physiologic cellular composition balances in the target tissue, and protect the immune system from suppression or hyper-activation.

Our computationally predicted approaches provide new platforms and resources for the formulation and analysis of testable hypotheses for CR in pro-cancer and cancer prevention mechanisms. Our models could be useful for further modelling and experimental validations of CR-response protecting metabolic reprogramming pathways. Further study is encouraging for the prospective utilization of CR-associated mechanisms in clinical oncology strategies.

## 5. Conclusions

- CR dramatically reduces immune responses, over-expresses the Paneth cell metabolic reprogramming pro-oncogenic genes forming aberrant networks, dysregulates tissue-specific epithelial proliferation and developmental processes, telomere maintaining processes, and response to chemical carcinogenesis;
- CR induces metabolic reprogramming processes driving pro-oncogenic mechanisms, cell cycle, and EMT pathways, and collectively increases the risk of malignancy;
- CR-induced *Rrm2, Lamc2, Fkbp5* and aberrant glutathione gene family activation coupled with *Sirtuin3* and *RNaseL* suppression could play tumorigenic roles in mucosa pathophysiology;
- Interferon-inducible gene family members on Chr11qB1.2 are suppressed by CR.

## Data Availability

The microarray datasets supporting the conclusions of this article are available in the ArrayExpress repository, https://www.ebi.ac.uk/arrayexpress/experiments/E-MTAB-6248. Accessed: 1 June 2018.

## Supplementary Materials

The following are available online at https://www.mdpi.com/article/10.3390/cancers13133180/s1. Supplementary Information containing Table S1: Functional enrichments common between human and mouse, Table S2: Cell cycle periodic genes regulated upon CR (CycleBase), Table S3: The 171 Immune system genes characterized using the Disease and Biological Function annotation tool from IPA, Table S4: CR-responded cancer stem cell related genes, Table S5: The 121 cancer-associated genes characterized using the Disease and Biological annotation tool from IPA, Table S6: Selected up-regulated oncogenes and down-regulated tumor suppressors upon CR, Table S7: Experimental validation targets, Table S8: IPA generated enriched canonical pathways, Table S9: IPA canonical pathway: Sirtuin Signaling Pathway, Table S10: Comparison of 18 microarray DEGs in duodenum mucosa with RT-PCR, Table S11: CR DEGs immune cell-type classification, Table S12: Gene symbols associated with multiple probe sets, Figure S1: Epithelial and immune system subcellular network, Figure S2: Epithelial and immune system CR DEGs single-cell expression profiling, Figure S3: NK, dendritic, phagocytic, and antigen presenting cell functional annotations network, Figure S4: Immune system, epithelial, and cancer-associated subset interaction networks, Figure S5: Liver tissue 3-week CR comparative analysis, Figure S6: Interferon-inducible GTPase paralogs, Figure S7: A model of CR-induced mucosal cells and pathway responses in DM. Supplementary Data containing Data S1: Mouse duodenum mucosa significant CR DEG subsets, Data S2: Gene enrichment and gene ontology analysis of 505 CR DEGs using DAVID Bioinformatics 6.7, Data S3: Cancer-associated gene expression patterns in CR response genes, Data S4: Key regulatory telomere maintenance genes are significantly altered, Data S5: The interferon-inducible GTPase (IFN-I) family on Chr 11 are suppressed by caloric restriction, Data S6: Drug targets in energy-responsive metabolic pathways, Data S7: Cancer-associated genes regulated upon CR in mice duodenum searched in PubMed for association with calorie restriction response studies. Additional details are included in Supplementary Methods and Discussion. Note: references [135,136,137,138,139,140,141,142,143,144,145,146,147,148,149,150,151,152,153,154,155,156,157,158,159,160,161,162] are not mentioned in main, only Suppl. Material.

## Abbreviations

Calorie restriction (CR), duodenum mucosa (DM), differential expressed genes (DEGs), immune system genes (ISGs), probe sets (p.s.), fold change (FC), false discovery rate (FDR), epithelial cell-enriched genes (ECG), cancer predisposition genes (CPGs), tumor suppressor genes (TSGs), transcription factor (TF), interferon (IFN), epithelial-mesenchymal transition (EMT), cancer stem cells (CSCs), duodenal intraepithelial lymphocytes (IEL), gastrointestinal (GI).
